# Anxiety and Depression during Transition from Hospital to Community in Older Adults: Concepts of a Study to Explain Late Age Onset Depression

**DOI:** 10.3390/healthcare3030478

**Published:** 2015-06-25

**Authors:** Aislinn F. Lalor, Ted Brown, Lauren Robins, Den-Ching Angel Lee, Daniel O’Connor, Grant Russell, Rene Stolwyk, Fiona McDermott, Christina Johnson, Terry P. Haines

**Affiliations:** 1 Department of Physiotherapy, School of Primary Health Care, Faculty of Medicine, Nursing and Health Sciences, Monash University—Peninsula Campus, Frankston, VIC 3199, Australia; E-Mails: aislinn.lalor@monash.edu (A.F.L.); lauren.robins@monash.edu (L.R.); angel.lee@monashhealth.org (D.-C.A.L.); 2 Monash Health, Allied Health Research Unit, Cheltenham, VIC 3192, Australia; E-Mails: ted.brown@monash.edu (T.B.); fiona.mcdermott@monash.edu (F.M.); 3 Department of Occupational Therapy, School of Primary Health Care, Faculty of Medicine, Nursing and Health Sciences, Monash University—Peninsula Campus, Frankston, VIC 3199, Australia; 4 Monash Health, Kingston Centre, Cheltenham, VIC 3192, Australia; E-Mails: daniel.oconnor2@monashhealth.org (D.O.); christina.johnson@monashhealth.org (C.J.); 5 School of Primary Health Care, Monash University, Notting Hill, VIC 3168, Australia; E-Mail: grant.russell@monash.edu; 6 School of Psychological Sciences, Monash University—Clayton Campus, Clayton, VIC 3800, Australia; E-Mail: rene.stolwyk@monash.edu; 7 School of Social Work, Faculty of Medicine, Nursing and Health Sciences, Monash University—Caulfield Campus, Caulfield East, VIC 3145, Australia

**Keywords:** hospitalization, older adult, anxiety, depression, community-living, post-discharge, health, wellbeing, falls

## Abstract

The transition between extended hospitalization and discharge home to community-living contexts for older adults is a critical time period. This transition can have an impact on the health outcomes of older adults such as increasing the risk for health outcomes like falls, functional decline and depression and anxiety. The aim of this work is to identify and understand why older adults experience symptoms of depression and anxiety post-discharge and what factors are associated with this. This is a mixed methods study of adults aged 65 years and over who experienced a period of hospitalization longer than two weeks and return to community-living post-discharge. Participants will complete a questionnaire at baseline and additional monthly follow-up questionnaires for six months. Anxiety and depression and their resulting behaviors are major public health concerns and are significant determinants of health and wellbeing among the ageing population. There is a critical need for research into the impact of an extended period of hospitalization on the health status of older adults post-discharge from hospital. This research will provide evidence that will inform interventions and services provided for older adults after they have been discharged home from hospital care.

## 1. Introduction

The transition between extended hospitalization and discharge to home for older adults is a critical period characterized by poor health outcomes, hospital re-admissions and gaps in healthcare service provision [1,2,3,4]. It is a period of change and adjustment for the patient, their carers and family, their social support network, and the health care system that provides services to them. This transition from hospital to community-living is important as individuals move from having their cares met for them to having to self-manage their health once home. The increased risk of functional decline and loss of independence are high and often permanent and are reflected in outcomes including increased falls, poor nutrition, functional decline, reduced activities of daily living (ADL) and depressed mood [3,].

Management of these problems can be difficult. Screening for anxiety or depression is not routinely employed at discharge from hospital despite symptoms of anxiety and depression being common at this point. It is unknown whether these symptoms resolve, persist or worsen over the months that follow. Indications from disease-specific research such as diabetes, age-related comorbidities, and Parkinson’s Disorder, suggest they are likely to persist and that these patients are not inclined to specifically seek mental health services to assist in their management [9,10,11,12,13]. Thus, anxiety and depression could be common mental health concerns that are not systematically being identified nor adequately managed despite a prolonged period of care for older adults within the health care system.

The aim of this current paper is to describe the concepts and design of a study aiming to:

- Assess the time-course of symptoms of anxiety and depression amongst older adults who have been discharged to the community following at least two weeks of hospitalization.
- Identify and understand inter-relationships between factors that may cause older adults to experience symptoms of anxiety and depression during the six months following an extended period of hospitalization.
- Develop a predictive index to identify older adults, at the point of hospital discharge, who are likely to experience clinically significant symptoms of anxiety or depression following discharge to the community.

## 2. Experimental Section

### 2.1. Study Design

This will be a mixed methods investigation comprised of an observational, prospective cohort study, and a qualitative investigation with thematic analysis from a phenomenological perspective.

### 2.2. Participants and Setting

Participants in the prospective cohort study will be adults aged 65 years and over who are transitioning home to community living following a period of extended hospitalization (two or more weeks). Study exclusion criteria will be cognitive impairment, discharge location to a residential aged care facility, length of stay in hospital of less than two weeks. Patients with cognitive impairment will be excluded due to the cognitive demands for completing the largely survey-based data collection approaches in this study. Cognitive ability will be determined by the investigator with the participant in person via completion of the 6-item Cognitive Impairment Test (6-CIT) [14]. Patients being discharged directly to a residential aged care facility will be excluded as they are returning to a care arrangement where many decisions regarding their health are determined by others on their behalf.

Participants will be recruited through Monash Health at the Kingston Rehabilitation Centre, Dandenong Hospital, and Casey Hospital, Melbourne, and Peninsula Health at the Golf Links Road Rehabilitation Centre, the Mornington Centre, and the Rosebud Rehabilitation Centre, Mornington Peninsula. These health services are suburban health networks in Victoria, Australia that provide tertiary level care to residents of that area.

Consecutive sampling of eligible patients from identified study wards will be employed until 300 participants have been recruited. In this study, the investigators seek to have 50% of the total sample being male and 25% identifying as being from culturally and linguistically diverse backgrounds. Use of quotas in this manner will ensure that sufficient data is available in the overall study sample so that if there are significant differences in outcomes between these sub-groupings they can be identified. There is evidence indicating that the incidence and response to mood disturbance is different between men and women, while people from culturally and linguistically diverse backgrounds have been found to encounter additional barriers in accessing health services which may affect their health outcomes following hospitalization.

Participants in the qualitative investigation will be drawn from the larger sample participating in the prospective cohort study. These participants will be purposively sampled on the basis of having experienced a “clinically significant” level of anxiety or depression symptoms during the six month period following their discharge from hospital.

### 2.3. Measurements

#### 2.3.1. Prospective Cohort Study Measurements

**Figure 1:**
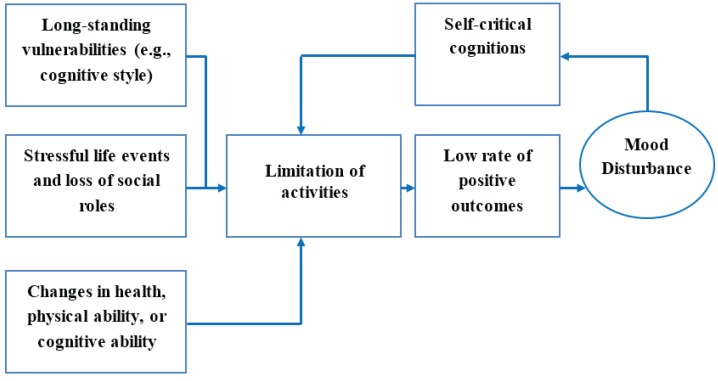
Behavioral model depicting onset and maintenance of depression in late life [15].

The investigators decided to capture a broad range of potential predictor/criterion variables in this study (given the relatively small amount of quantitative information currently available) on factors found to be associated with mood disturbance, particularly late age onset depression. As a starting point, the “Behavioral model depicting onset and maintenance of depression in late life” [15] was used to guide the selection of data-gathering tools (refer to Figure 1). This model proposes an explanation for the development of late age onset depression and anxiety and depicts various domains related to aging including: the interaction between longstanding vulnerabilities (e.g., genetic factors) and stressful events that are more likely in later life (e.g., spousal bereavement, loss of roles); in addition to biological factors (physical or cognitive) [15]. This interplay can limit both the capacity and the participation levels of an older adult and lead to reduced activities. Potential further compounding factors include self-critical cognitions, low rate of positive outcomes and mood disturbance (e.g., a depressed person may be overly critical of their engagement in an activity, feel they performed poorly, and this then leads them to further reduce their participation in activities they previously engaged in). Negative reinforcement can occur of this behavior as future attempts to engage in activities may be considered to result in failure and therefore a feedback loop of negative cognitions is established. This study will operationalize the three input domains and the negative feedback loop as the output feedback loop domain.

To measure these three input and one output domains, a suite of measures across a range of fields were pilot tested and subjected to review by a Project Reference Committee. Pilot testing of the initial baseline questionnaire was conducted with five non-hospitalized, community-dwelling older adults that are representative of our target population. Feedback we received from our consumers was that the survey was overly burdensome and that there were several items that appeared repetitious that could be removed and this led to modification of some measures. The Short Geriatric Depression Scale (GDS15) [16], Geriatric Anxiety Inventory (GAI) [17], EuroQol-5 Dimensions-5 Levels (EQ-5D-5L) [18], PhoneFITT [19], Epworth Sleepiness Scale (ESS) [20], Pittsburgh Sleep Quality Index (PSQI) [21], and the Death Anxiety Questionnaire (DAQ) [22], along with items relating to current falls and exercise program adherence were used to capture the output feedback loop domain. The Intrinsic Spirituality Scale (ISS) [23], DAQ, Pain Attitudes Questionnaire (Revised; PAQ-R) [24], Brief Resilient Coping Scale (BRCS) [25], and Ten-Item Personality Inventory (TIPI) [26] along with single item questions relating to demographic data, housing and financial situation, and medical history were used to capture the long-standing vulnerabilities domain. The Friendship Scale [27] and Lubben Social Network Scale Abbreviated (LSNS-6) [28] along with single item questions relating to services received, caring or volunteering roles, computer use, transport options and stressful life events were used to capture the stressful life events and loss of social roles domain. The Controlled Oral Word Association Test—Semantic (category) version (COWAT-S) [29], Color Trails Test (CTT) [30], and Urogenital Distress Inventory (UDI-6) [31], along with single item questions relating to demographic data and health and wellbeing history were used to capture the changes in health, physical activity and cognitive ability domain. The variables and domains that are measured by the tools mentioned above are summarized in Table 1. This table also highlights at which time points during the study these measures are proposed to be utilized. The measures selected within each domain are now presented and highlight any modifications that were made as a result of the pilot and review process. Appendix Table A1 has also been provided summarizing the psychometric properties of the summarized measures.

**Table 1:** Summary of the key domains assessed via questionnaire in the study of older adults.

Domain	Questionnaire Data (Measurement Tool)	Measurement Points							
		R	B	1	2	3	4	5	6
Output feedback loop	Depression symptoms (GDS15, EQ-5D-5L)	X	X	X	X	X	X	X	X
	Anxiety symptoms (GAI, EQ-5D-5L)	X	X	X	X	X	X	X	X
	Physical capacity and participation (PhoneFITT)	X	X	X	X	X	X	X	X
	Quality of life (EQ-5D-5L)					X			X
	Falls		X	X	X	X	X	X	X
	Sleepiness and sleep quality (ESS, PSQI)	X	X			X			X
	Perception of death * (DAQ)		X						
	Exercise program		X			X			X
Long-standing vulnerabilities	Gender		X						
	Culturally and Linguistically Diverse (CALD)		X						
	Marital status		X						
	Housing situation		X						
	Financial situation		X						
	Primary occupation		X						
	Education level		X						
	Existing chronic conditions *		X						
	Religiosity/spirituality (ISS)		X						
	Perception of death * (DAQ)		X						
	Pain and stoicism (PAQ-R)		X						
	Resilience and coping style (BRCS)		X						
	Personality (TIPI)		X						
Stressful life events and loss of social roles	Services received		X						
	Social isolation (LSNS-6, Friendship Scale)		X			X			X
	Computer use		X			X			X
	Driving/transport		X			X			X
	Carer/volunteering		X						
	Stressful life events		X			X			X
Changes in health, physical ability, or cognitive ability	Cognition (COWAT-S, CTT)		X			X			X
	Vision and visual aids		X						
	BMI		X						
	Falls history	X							
	Physical capacity and participation * (PhoneFITT)	X	X	X	X	X	X	X	X
	Continence (UDI-6)		X			X			X
	Reason for hospital admission		X						
	Existing chronic conditions *		X						
	Nutrition		X						
	Caffeine intake		X			X			X
	Alcohol intake		X			X			X
	Smoking intake		X			X			X
	Health professional consultations		X			X			X
	Medication		X			X			X

Note: * denotes questionnaire data relevant to two or more domains; B: Baseline questionnaire; R: Retrospective questionnaire; 1: 1 month questionnaire; 2: 2 month questionnaire; 3: 3 month questionnaire; 4: 4 month questionnaire; 5: 5 month questionnaire; 6: 6 month questionnaire; GDS15: Short Geriatric Depression Scale; EQ-5D-5L: EuroQol-5 Dimensions-5 Levels; GAI: Geriatric Anxiety Inventory; ISS: Intrinsic Spirituality Scale; DAQ: Death Anxiety Questionnaire; PAQ-R: Pain Attitudes Questionnaire (Revised); BRCS: Brief Resilient Coping Scale; TIPI: Ten-Item Personality Inventory; LSNS-6: Lubben Social Network Scale Abbreviated; COWAT-S: Controlled Oral Word Association Test—Semantic (category) version; CTT: Color Trails Test; UDI-6: Urogenital Distress Inventory; ESS: Epworth Sleepiness Scale; PSQI: Pittsburgh Sleep Quality Index.

#### 2.3.2. Initial Cognitive Screen

An initial cognitive screen is proposed prior to participants being recruited for the study in order to ensure sufficient cognitive ability to partake in the study over the six months. The 6-CIT [14], also known as the Short Orientation-Memory-Concentration Test, is a brief cognitive test used in primary care and involves three orientation items.

#### 2.3.3. Output Feedback Loop Domain

This domain contains several different measures that address aspects of a person’s health and activities that form part of a negative feedback loop thought to culminate in mood disturbance. Previous authors proposed that both the “lack of opportunity for positive outcomes and the aversive experience of self-critical cognitions may intensify and maintain a depressive state” [15]. In addition the investigators hypothesize that falls, sleep disturbance, and loss of physical capacity are additional compounding factors that will be important factors within this feedback loop for older adults. Symptoms of depression will be measured using the GDS15. The GDS15 [16] is a shortened version of the original 30 item Geriatric Depression Scale (GDS). The GDS was created for use in geriatrics as its items were based on characteristics of depression in the elderly [32]. The GDS15 is a 15 item yes/no questionnaire devised to detect depression in later life, specifically within the older population (65 years and over). To determine clinically significant symptoms of depression, a cut-off of six or more was used, in accordance with recommendations by the original authors [16,33]. Symptoms of anxiety will be measured using the GAI. The GAI is a 20-item agree/disagree questionnaire that was developed as a simple instrument to allow measurement of anxiety symptom severity in older adults in varied settings [17]. While a score of eight correctly identified 78% of patients with any anxiety disorder in a group of older adults with psychiatric disorders, a cut-off score of nine or greater was used to determine clinically significant symptoms of anxiety, as per original author suggestion [17]. The EQ-5D-5L is a 5-item measure that will assess depression and anxiety symptoms and quality of life in the Output Feedback Loop.

Physical capacity and participation will be assessed with the Phone-FITT. This measure is a brief physical activity interview for use with older adults [19]. It was designed to measure dimensions of physical activity including: Frequency, Intensity, Time and Type (FITT) as identified as the most familiar dimensions required in the context of aerobic endurance training by the American College of Sports Medicine [34]. Activities included are those prevalent among older Canadians (where the scale was developed) and those that have demonstrated importance in falls prevention (e.g., balance and strengthening exercises) [19].

A participant’s perception of death will be assessed via the DAQ within this study. The DAQ is a 15-item, three point scale (“not at all”, “somewhat”, “very much”) reported to assess the specific fears that individuals may have when thinking about death or dying [22]. The 15-items are classified across five factors: fear of the unknown; fear of suffering; fear of loneliness; fear of personal extinction; and, unclassified. Items were removed from the DAQ on the judgment of investigators as they were deemed as being least relevant to the overall study aims and in an attempt to reduce overall respondent burden. Daytime sleepiness and overall sleep quality will be assessed in this domain via the ESS and PSQI. The ESS is an 8-item subjective measure of daytime sleepiness [20,35]. Using a 4-point Likert scale (0 = “No chance” to 3 = “High chance of dozing”) respondents rate their likelihood of falling asleep or dozing during eight common situations in life (e.g., sitting and reading). A score greater than 10 (out of a possible 0–24) is considered clinically significant in relation to daytime sleepiness [20]. The PSQI is a 19-item measure of retrospective sleep quality and disturbances relating to the individual’s recollection of night-time sleep quality over the past month [21,36]. The PSQI yields scores across 7 equally weighted component domains including: (1) Subjective Sleep Quality; (2) Sleep Latency (time it takes to fall asleep); (3) Sleep Duration; (4) Habitual Sleep Efficiency (ratio of total sleep time to time in bed); (5) Sleep Disturbances; (6) Use of Sleep-Promoting Medication (prescribed or over-the-counter); and, (7) Daytime Dysfunction. The PSQI uses a combination of open-ended questions and a 4-point Likert scale (0 = “Not during the last month” to 3 = “Three or more times a week” in relation to problem frequency; or 0 = “Very good” to 3 = “Very bad” in relation to overall sleep quality). Overall points are summed (range 0 to 21) where a higher overall score (Global Score) indicates poorer sleep quality. Component scores range from 0 to 3 and are summed to obtain the Global Score. A cut-off score of >5 was empirically derived and distinguishes poor sleepers from good sleepers [21].

Falls in the study will be assessed through subjective recall over the past month. Participants are provided with the World Health Organization definition of a fall. Evidence from a systematic review of falls methodology has shown that there is no “gold standard” for documenting falls, however, if retrospectively collected it is recommended that details are ascertained at least once a month (as is proposed in the present study) to reduce limitation of recall bias [36].

#### 2.3.4. Long-Standing Vulnerabilities Domain

This domain reflects background traits and experiences that are thought to predispose, or protect against, older adults from developing late age onset depression. Potential indicators of long-standing vulnerabilities included in the initial baseline questionnaire for this study consist of information regarding the person’s home environment (e.g., natural lighting); socioeconomic status (e.g., financial, education and housing situation); existing chronic conditions; religiosity or spirituality; perception of death (using the DAQ previously mentioned); pain and stoicism; resilience and coping style; personality; additional demographic items (e.g., gender, marital status); and a participant’s Culturally and Linguistically Diverse (CALD) status. Previous research has demonstrated that people from CALD backgrounds are likely to have experienced, and attempting to recover from, loss, grief, torture, trauma, and the obstacles of resettlement [37,38]. Additionally, they may lack access to mental health services due to stigma, language difficulties, or unfamiliarity with the health system of Australia, thereby placing greater demands on them to cope with limited appropriate support regarding their mental health. Participants in this study will be classified as being from a CALD background if they answer “Yes” to two of the three following questions: (1) Were you born in a country other than Australia? (2) Do you speak a main language other than English at home? (3) Do you identify with a specific cultural group (other than Australian) or as an Indigenous Australian or Maori?

A participant’s religiosity or spirituality will be assessed via the ISS, which is a 6 item measure designed to assess the degree to which an individual’s spirituality functions as a “master motive” beyond a religious framework [23]. During piloting of items, only two of the six items in the ISS were considered to have face validity while the other four items were ambiguous. Therefore, only these two items have been included in the study questionnaire. Additionally, the PAQ-R will be included to measure pain and stoicism. The PAQ-R is a 24 item, 5-point rating scale (1 = “Strongly disagree” to 5 = “Strongly agree”) of the attitudes of stoicism and cautiousness individuals may have towards perception and reporting of pain symptoms [24,39]. Within the proposed measure (PAQ-R) five of the possible 24 items, relating to the Stoic-Fortitude sub-scale, were identified as being appropriate for inclusion in order to reduce respondent burden.

Resilience refers to the “dynamic process that results in adaptation in the context of significant adversity” [40] (p. 60). The BRCS is a 4-item measure that uses a 5-point rating (1 = “Does not describe me at all” to 5 = “Describes me very well”) designed to measure an individual’s tendencies to cope with stress in a highly adaptive manner (an individual’s competence of daily skills to meet everyday living demands) [25]. Lastly, personality will be briefly assessed via the TIPI. This measure is a 10 item personality scale utilizing a 7-point Likert scale (1 = “Disagree strongly” and 7 = “Agree strongly”) [26]. The TIPI includes the identified “*Big Five*” dimensions of personality: “extraverted”, “agreeable, warm”, “conscientious”, “emotionally stable”, and “open to new experiences”. The TIPI was developed for use in research screening where personality is not the primary topic of interest (as is the case for this project) and where brevity is required to reduce respondent burden [26].

#### 2.3.5. Stressful Life Events and Loss of Social Roles Domain

This domain reflects events, largely external, that may impact on an older adult’s propensity to develop late age onset depression; some may be sudden (e.g., loss of partner), while other events may take place over an extended period (e.g., loss of social role). This domain will be assessed via the LSNS-6 and Friendship Scale in addition to various social role factors. The LSNS-6 is an abbreviated version of the Lubben Social Network Scale (LSNS) to lessen respondent burden, and was produced to screen for social isolation [28]. The LSNS was specifically developed for use among older adult populations [28]. It uses a two factor structure (family and friends) to measure perceived social support from family (three items) and friends (three items) [41]. The LSNS-6 uses a six-point scale of the number of family or friends within the past month that the person reports seeing or hearing from in relation to the item asked (0 = “none” through to 5 = “nine or more”). The Friendship Scale is a short 6-item scale with a 5-point scale (“Almost always” to “Not at all”) devised to assess social isolation in older adults [27]. Each item is scored 0–4 with a possible range of 0–24 overall. Scores between 0 and 15 indicate low friendship acuity, 16 and 18 moderate friendship acuity, and 19 and 24 high friendship acuity [27]. Four items were removed from the LSNS-6 for inclusion within this study as they overlapped with the Friendship Scale.

#### 2.3.6. Changes in Health, Physical Ability, or Cognitive Ability Domain

This domain reflects changes to the internal health and capacity of the older adult to function. Cognition will be assessed via the COWAT-S and the CTT, and continence via the UDI-6. Additional items will assess a participant’s Body Mass Index (BMI); reason for hospital admission and existing chronic conditions; intake of caffeine, alcohol, and/or tobacco; their connection with a regular general practitioner (GP); and consultations within the past month with their GP or other health professional. The COWAT-S is a category fluency task to assess executive functioning, semantic knowledge and memory retrieval ability [29]. Category fluency tasks require an individual to name as many animals (or supermarket items or similar) as possible within one minute from memory. The number of category items reported, repeated words and words not pertaining to the category are all recorded. Category fluency is believed to be appropriate for use with individuals across various backgrounds to allow for demographic correction relating to age, education and ethnicity. Norms for the COWAT-S have been developed to adequately address ethnicity, education and age [29]. The CTT consists of two timed trail tests where individuals are required to connect circles numbered 1 through to 25 in sequence with a pencil as fast as possible [30]. For the CTT 1 trail, the respondent has one set of numbers to connect (1–25). For the CTT 2 trail, the respondent is presented with duplicate colored numbers within the range (1–25). Participants are required to rapidly connect these in sequence, while alternating between pink and yellow colored circles. Both trail tests assess visual scanning, graphomotor skills, sustained visual attention and allow the assessor to also obtain information regarding eye-hand coordination speed and information processing speed as the respondent completes the trails [30]. To reduce cultural and linguistic bias, the CTT uses no letters and can be administered verbally or non-verbally through demonstration [30,42]. However, participants do need to be able to recognize Arabic numerals (1 to 25) and distinguish colors pink and yellow [30,43]. The time to complete both trails is recorded in seconds with errors, near misses, and prompts also recorded.

The UDI-6 is a 6-item, 4-point measure designed to assess the symptom distress and life impact of urinary incontinence [31]. It was developed from the Urogenital Distress Inventory—Long Form which consists of 19 items [44]. Respondents are asked whether they currently experience, and how much they are bothered by (0 = not at all, 1 = slightly, 2 = moderately, 3 = greatly), various urinary incontinence issues (e.g., urinary leakage related to the feeling of urgency). For the present study, only three items of the UDI-6 (2, 3, and 4) have been included along with an additional item (“problems with your bowels, like constipation or diarrhea”) which was determined following piloting of the items. During piloting respondents indicated that bowel issues, not just bladder concerns, impacted on their likelihood of leaving their home or socializing with family or friends.

Additional questions will be asked at the baseline assessment phrased about the patient’s current condition and questions phrased about the patient’s recollection of their pre-morbid condition. Table 1 outlines the time periods for each measure and additional questions across the eight time periods (e.g., R: Retrospective, B: Baseline, 1–6: 1 month to 6 months). For example, a current condition question: “If you were to try today, could you walk up and down stairs without a handrail or assistance from someone else?” for pre-morbid condition would be rephrased: “Prior to coming into hospital, if you were to try, could you walk up and down stairs without a handrail or assistance from someone else?”

#### 2.3.7. Qualitative Measurements—Semi Structured Interview

At completion of the 6 month follow-up, the investigators will specifically target participants that exhibit clinically significant symptoms of depression and/or anxiety and invite them to participate in semi-structured interviews. The interviews will be aimed at eliciting their narrative account of how they experienced their transition from hospital, their time course of symptoms of depression and anxiety, their explanation as to why they feel they experienced these symptoms, and their account of any strategies they used to try and manage this problem. A list of the questions and description of techniques that will be used to facilitate the discussion can be viewed in the Appendix Box A1 and Appendix Table A2.

### 2.4. Procedure

Potential participants will be identified by screening of ward discharge planning lists on the targeted hospital wards. Discharge dates are tentatively set within 48 h of admission, however, need to be confirmed at least 24–72 h before the actual discharge date. Those appearing to meet the study inclusion criteria will be screened by project research personnel to confirm eligibility and then be approached for consent to participate. Those consenting will have the baseline assessment completed within 48 h to the time of discharge, of the planned discharge date. The baseline assessment will include measurements as previously outlined.

Once the participant has been discharged from hospital, they will be asked to undertake a telephone interview follow-up asking about a subset of domains for the 1, 2, 4, and 5 month assessments. These domains were selected to be examined as they were either the primary study outcomes which the investigators are trying to describe the time-course to address research aim 1, or they were central to the limitation of activities model for explaining development of mood disturbance in this population. The follow-up assessments being undertaken at 3 and 6 months post-discharge will be undertaken using a face-to-face interview approach. Accredited language interpreters will be used in both studies when required.

### 2.5. Analysis

#### 2.5.1. Aim 1. Time Course Symptomology

The mean and standard deviation in GDS15 and GAI scores will be plotted over the 6 month transition period. The influence of time since discharge on GDS15 and GAI scores will be examined using a multi-level generalized linear model with assessment nested within participant in the random effects part of the model, while time since discharge will be treated as a fixed factor. Data will also be visually inspected to determine if there are common patterns within the time-course in levels of these symptoms. A random sample of 50 participants will be selected to identify these patterns that will characterize the time-course of symptomology (e.g., participants who experience a short spike in symptoms of depression which then resolves). The remainder of the sample will be used to estimate the proportion of participants that fit into each of these categories. Binomial 95% confidence intervals will be used to represent the uncertainty in these estimates. Two assessors will classify each participant’s time course pattern and the agreement between these assessors will be examined using Cohen’s Kappa.

#### 2.5.2. Aim 2. Factors That Increase Symptoms of Anxiety or Depression

A mixed methods analysis approach will be used to address this aim. A thematic analysis of qualitative data captured at the six month assessments will first be used to identify the factors participants’ identified as causing their symptoms of anxiety or depression to increase. The interaction and effect modification that may exist between these individual factors will also be a point of focus for the analysis such that a model that explains the worsening of symptoms of depression and anxiety can be developed. This will be our preliminary explanatory model. The credibility and neutrality of this model will be examined by testing the explanatory power of this model using the quantitative dataset. It is anticipated that most, if not all, of the factors identified in the qualitative analysis will map onto domains being measured within our prospective cohort study dataset as the investigators used a leading model of development of late-age onset depression to guide the selection of these quantitative variables. The transferability of this model will be able to be tested using the quantitative data by examining whether the explanatory power of the model is consistent across male/female groupings and those from CALD/non-CALD backgrounds.

This quantitative dataset will be used to then test the preliminary explanatory model. Latent growth curve modelling will be used to examine the strength of associations between the factors included in our preliminary explanatory model and the study outcomes of depression measured using the GDS15, anxiety measured using the GAI, and a combination of the two measured using the EQ-5D-5L anxiety/depression item. The model fit will be refined by removing factors that do not have a significant association within the model. Factors not included in the original preliminary explanatory model will be added as it is possible that patients may not be aware of factors that were important in the development of their symptoms (e.g., the unknown self). Those that have significant associations within the model will be retained culminating in our definitive model to explain development of symptoms of anxiety and/or depression in this population.

#### 2.5.3. Aim 3: Predictive Index Development

**Figure 2:**
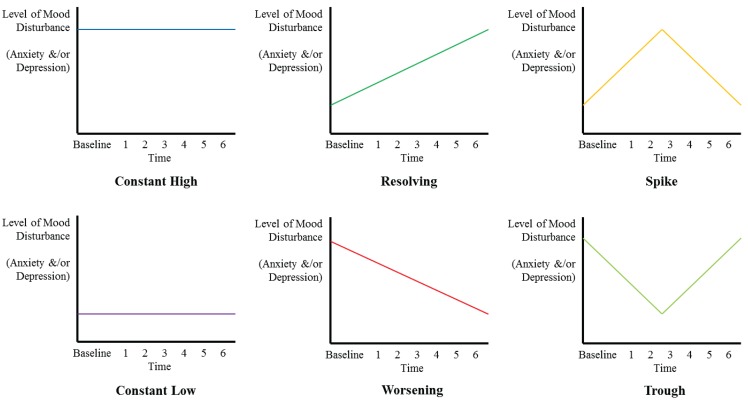
Anticipated “patterns” of anxiety or depression.

Participants will be categorized as to the “pattern” of anxiety or depression they have exhibited based on analyses to address aim 1. The investigators anticipate that participants will fit into one of six categories as displayed in Figure 2. Additional categories may be developed as are emergent from the data. These categorizations will be used as dummy dependent variables in logistic regression models developed to predict membership of these categories based on information captured at the baseline assessment. Data from 150 randomly selected participants will be used to develop these predictive models while data from the remaining participant cohort will be used to test the accuracy of these models. Sensitivity, specificity, positive predictive value, negative predictive value, calibration, and the Youden Index will be used to describe the accuracy of these models [45].

#### 2.5.4. Sample Size

A sample size of 150 participants being analyzed in the development or the validation analyses of the predictive index will provide 80% power (assuming α = 0.05) of identifying a sensitivity or specificity of 0.65 as being significantly more accurate than random chance (a sensitivity or specificity of 0.50) [46,47].

## 3. Discussion

This study will be the first to prospectively investigate the inter-relationships between factors that may cause older adults to experience symptoms of depression and anxiety during the six months following an extended period of hospitalization. Individual outcome data will be collected over the six month time period to assist with assessment of the time-course of symptoms of anxiety and depression that older adults may experience post-discharge following at least two weeks of hospitalization.

The investigators anticipate that the development of a predictive index to identify older adults who are likely to experience clinically significant symptoms of anxiety or depression following discharge to the community will be of clinical importance. This will allow interventions to be targeted to those who need them most. Identification of factors that precipitate development of symptoms of depression or anxiety may also aid development of interventions targeted at these factors. This hopefully will also assist with reducing re-admissions to hospital, and impact on the functional outcomes of this patient group upon returning home. This study will also assist with understanding the explanatory power of the “Behavioral model depicting onset and maintenance of depression in late life” [15].

This study will provide important information regarding both causative mechanisms (such as social isolation, lack of resilience, and changes in sleep quality) and impacts of anxiety and depression amongst older adults []. It will enable health services to better address these issues and potentially break the vicious cycle represented in the Behavioral Model by the output feedback loop. Specifically, this study will identify patterns in symptoms of anxiety and depression and their relationship to physical capacity and falls which could be targets for intervention. It will enable early identification of those at risk of experiencing depression during the transition period, and will identify those unlikely to otherwise access mental health services to assist with management of their depressive symptoms. It will also permit exploration of how depression interacts with other geriatric conditions such as sleep problems and social isolation, while identifying opportunities for health care service delivery reform to enable more comprehensive management of the older adult who has recently had an extended period of hospitalization.

This study has several limitations that require acknowledgement. Firstly, due to the age of participants to be recruited and/or the length of time participants are to be engaged with the study, there is the potential for dropouts or participants to die. Participants are required to complete a baseline questionnaire prior to their discharge to community-living which takes approximately one hour to complete. Additional follow-up questionnaires will take between 10 and 30 min to complete depending on the month of follow-up. This time burden may affect initial recruitment and retention as well as potentially impact on recruitment for participants to take part in the qualitative semi-structured interview (whereby some potential participants may decline to participate in the second part of the study having completed the first six months of follow-up). Some tools included do not have research to establish whether they are able to detect change over each month or over a 3 month time frame.

Another limitation of this study was that the investigators made modifications to the content of previously developed and validated measures. This was necessary to minimize duplication and overall participant burden in completing the questionnaires as the investigators were concerned that a more burdensome survey would lead to greater participant attrition. [54,55] This means that analyses will be unable to use pre-existing summative scale scores in the analyses where scales have been modified. Instead, scores from individual items and/or factor analysis procedures will need to be used when building latent growth curve models.

One strength of this study lies in its prospective design with repeated measurement of constructs of interest. Information will be gained directly from participants rather than as a result of observations from a health professional allowing for participants to provide information from their own perspective. This is particularly important as participant-centered information can sometimes be lost in quantitative research. Furthermore, the qualitative semi-structured interviews will drive the analysis of quantitative data. Future work that may emanate from this research should focus on development of interventions targeting factors found to precipitate symptoms of depression and/or anxiety in this population.

## 4. Conclusions

This study will provide important insights into the health and wellbeing of older adults while they transition to community-living following an extended period of hospitalization. This study will fill an important gap in our understanding of depression and anxiety symptoms and the associated comorbidities in this population. It will further provide a unique contribution to the existing research body of knowledge due to the unique prospective study design that incorporates both quantitative and qualitative data collection methods. This mixed methods design allows the patient reported experience of these issues to drive the quantitative data analysis, and be central to the overall study findings.

## Appendix

**Table A1:** Psychometric properties of proposed tools to be included.

Measure	Reliability/Validity	Sample Item
Short Geriatric Depression Scale (GDS15)	The GDS15 has a high level of internal consistency (Cronbach’s α = 0.80) [56] and strong sensitivity (81.3%) and specificity (78.4%) [57]. Additionally, the efficiency (fraction correctly identified) of the GDS15 is significantly higher than the GDS (77.6% *vs.* 71.2%, Chi² = 24.8, *p* < 0.0001) and the clinical utility of the GDS15 was rated as “good” for screening (UI—0.75) [57].	Individuals are asked to choose the best answer for how they have felt over the past week, e.g., “Are you basically satisfied with your life?”
Geriatric Anxiety Inventory (GAI)	The GAI has well established psychometric properties in various population groups within the older aged [58], with high test-retest reliability (*R* = 0.91) and inter-rater reliability (*R* = 0.99) [17] and demonstrated sensitivity (85.7%) and specificity (78.0%) [59].	Individuals are asked to choose the best answer for how they have felt over the past week, e.g., “I worry a lot of the time”.
6-item Cognitive Inventory Test (6-CIT)	It takes less than 5 min to complete (mean 2.5 min) and has demonstrated high correlation (*R*^2^ = 0.911) with the Mini-Mental State Examination [60,61,62]. Recent evidence has highlighted the advantages of using the 6-CIT over the MMSE in hospital settings [62]. It has also demonstrated good sensitivity and specificity of 78.57% and 100% (cut-off 7/8) for detecting mild dementia and when compared to the Mini-Mental State Examination (90% and 96%, respectively) [60,62]. It is recognized for being culturally unbiased and has further demonstrated not to be sensitive to educational level, nor require advanced language skills [62]. The 6-CIT has limited validation data available although stable reliability (*test-retest* immediate: *Pearson’s r* = 0.68; *test-retest* delayed: *Pearson’s r* = 0.74) has been reported [63].	Individuals are asked to “Count backwards from 20-1”.
Phone-FITT	Preliminary evidence demonstrates substantial test-retest reliability (95% CI, intra-class correlation coefficients 0.74–0.88; Spearman’s rho = 0.29–0.57), in addition to concurrent, convergent and discriminant validity [19].	Individuals are asked initially to answer Yes/No as to whether they completed an activity (e.g., Light housework such as tidying, dusting, laundry, or ironing). If the individual answers yes, they are then asked “How many times in the past week did you complete this activity?” Individuals are also asked “About how much time did you spend on each occasion completing this activity?”
2.3.19. EuroQol-5 Dimensions-5 Levels (EQ-5D-5L)	The EQ-5D-5L was recently developed following revision of the EQ-5D-3L to improve sensitivity and reduce possible ceiling effects previously found in the EQ-5D-3L. [18] Recent research has supported the revised version for sensitivity [64].	Measures 5 dimensions of health including: mobility, self-care, usual activities, pain/discomfort, and anxiety/depression across a five point scale (0 = “No problems” to 5 = “Extreme problems”) [18].
Intrinsic Spirituality Scale (ISS)	The overall measure reports strong internal consistency (Cronbach’s α = 0.96), strong reliability (0.80), and strong construct validity (*r* = 0.91, *p* < 0.001) [23].	The ISS uses a ranking scale from 0 to 10 where 0 = “Plays absolutely no role” to 10 = “Is always the over-riding consideration”. Individuals are asked to rank themselves in response to each item (e.g., “When I am faced with an important decision my spirituality…”).
Death Anxiety Questionnaire (DAQ)	Initial research suggests discriminative validity of the items, construct and concurrent validity of the scale as a whole, and applicability over a broad age range ranging from 30 to 82 years [22]. Subsequent research has indicated excellent internal consistency (Cronbach’s α = 0.90) and strong factor structure [65].	Individuals are asked to respond either “not at all”, “somewhat”, or “very much” in relation to each item (e.g., “Do you worry about dying?”)
Pain Attitudes Questionnaire (Revised) (PAQ-R)	Previous evidence suggests chronic pain sufferers attempt to preserve their self-esteem and maintain acceptance socially by exhibiting stoicism and reduce negative (expected or real) social consequences of disclosure [66,67].	Individuals are asked to select the best answer for them (e.g., “I do not see any good in complaining when I am in pain”) [39].
Brief Resilient Coping Scale (BRCS)	Initial evidence exists relating to adequate reliability of the tool (Cronbach’s α = 0.69; test-retest correlation = 0.68–0.71, *p* < 0.001) and validity (*r* = 0.37, *p* < 0.01), given the brevity of the measure [25,67,68]. Preliminary evidence demonstrates that the BRCS is a reliable and valid measure of resilient coping in non-English speaking elderly populations [69].	Individuals are asked to select the extent to which they agree to each of the statements (e.g., “I tend to bounce back quickly after hard times”).
Ten-Item Personality Inventory (TIPI)	Initial evidence reports adequate psychometric levels for the tool. [26]	Individuals are asked to rate how they perceive themselves across various personality traits (e.g., “I see myself as: extraverted/enthusiastic”).
Lubben Social Network Scale Abbreviated (six items; LSNS-6)	The two factor (family and friends) structure was confirmed across three European community samples and loaded highly on each factor indicating strong construct validity. [70] The LSNS-6 has high levels of internal consistency (Cronbach’s α = 0.83) and correlations with criterion variables. [70]	Individuals are asked to rate, where 0 = None and 5 = Nine or more, “considering the people to whom you are related either by birth or marriage, how many relatives (including spouses, partners, children, *etc*.) do you see or hear from at least once a month?”
Friendship Scale	Developed in Australia, the Friendship Scale comprises six of the seven identified dimensions that are believed to contribute to social isolation or social connectedness. [27] While the Friendship Scale has limited related publications at present, initial evidence suggest it has excellent internal structures (CFI = 0.99, RMSEA = 0.02), strong reliability (Cronbach’s α = 0.83), and concurrent discriminant validity suggesting sensitivity to known social isolation correlates. [27]	Individuals are asked to rate, on a 5-point scale from “Almost always” to “Not at all”, over the past 4 weeks “It has been easy to relate to others”
Color Trails Test (CTT)	Research has been conducted comparing the utility of the CTT to three other tests for assessing executive functioning in older adults and was found to be the highest loading for the executive function domain (factor loading = 0.57). [71] Further evidence also suggests that the CTT is appropriate for cross-cultural and clinical assessment of mental processing speed, sequencing, and visual scanning in non-English-speaking adults and adults with limited education. [72]	N/A
Urogenital Distress Inventory (UDI-6)	Research has demonstrated that the UDI-6 has strong psychometric properties (Cronbach’s α = 0.93) and is considered more useful in clinical and research settings. [31] High internal consistency (Cronbach’s α = 0.74) and test-retest reliability (Spearman’s rho = 0.99, *p* <0.001) was demonstrated with a sample of 302 Turkish speaking women with urinary issues. [73] Furthermore, while predominantly utilized with women, the UDI-6 has been used in studies with both males and females and identified high levels of distress relating to urinary issues in males that had not previously been detected. [74]	Individuals are asked whether they currently experience: “Urine leakage related to the feeling of urgency” (Yes or No).
Epworth Sleepiness Scale (ESS)	The ESS has high internal consistency (Cronbach’s α = 0.88) and test-retest reliability (*r* = 0.82) [20,35,74,75,76].	Individuals are asked to choose the most appropriate response, on a 4 point scale where 1 = would NEVER doze or sleep to 4 = HIGH chance of dozing or sleeping, for various situations (e.g., “Sitting and reading”).
Pittsburgh Sleep Quality Index (PSQI)	Initial development of the PSQI was conducted with patients with major depression and patients with a sleep disorder. [21] It has subsequently become a widely used, recognized and validated tool for assessing sleep quality with participants presenting with a variety of medical diagnoses. [77,78] More recently, the PSQI has been further validated for use with community-dwelling older men and older women. [79,80] Adequate internal consistency was reported for total PSQI scores (Cronbach’s α = 0.69). Previous research also demonstrated good internal consistency (Cronbach’s α = 0.78) for the PSQI in both black (*n* = 306) and white (*n* = 2662) community-dwelling women aged 70 years and over. [79] Adequate test-retest reliability (0.85), strong criterion validity, and responsiveness to have also been established for the PSQI. [21,81] The global score has a strong diagnostic sensitivity (89.6%) and specificity (86.5%). [82]	Individuals are asked to answer various items relating to their usual sleep habits during the past month. (e.g., “During the PAST month, what time have you usually gone to bed at night?”)

Box A1 Qualitative Questions and Technique The second part of this study involves selected participants engaging in a qualitative semi-structured interview to relate their experiences of their transition home from extended hospitalization and the implications for their mental health and wellbeing. The purpose of the qualitative interviews is to capture the participant’s narrative account of why they felt they experienced mood disturbance during their transition from hospital. The question set was developed following a meeting with the project reference group consisting of health practitioners, service providers and consumer advocacy group representatives. A semi-structured interview will be undertaken using the question set in Table A2 as a guide for each interview.

**Table A2:** Proposed question set for the qualitative semi-structured interviews at completion of the overall study.

Area/Construct	Potential Questions
**Build rapport with the participant**	How has your week been? How are you feeling? How do you feel about us doing this interview? Did you have thoughts about what we are going to talk about today? Activity: Have “visual” graphic of their GDS15 and GAI results over the 6 month period to aid participant to visualize their change in symptoms of depression and anxiety
**Establish participant’s expectation for the interview**	Looking back over the past 6 months are you at where you expected? What is it that hasn’t met your expectation(s)? Did you even consider what you would expect? Do you think you were prepared for what has happened over the past 6 months? What were you not prepared for?
**Narrative—establish the participants experience**	1. Would you say you’ve had some tricky times?/Would you say you’ve had some up’s and down’s? 2. We’ve noticed that during the study you’ve ...(refer to visual graph of GDS15 over the 6 month period) Can you tell us about this...? * Was there something happening at that point of time for you? * Did you do something at that time to assist you with coping? * Was there a particular strategy that worked for you?
**Functional Decline/Falls Experience of the participant and how this relates to their symptoms of depression or anxiety**	How would you feel about asking your GP about this...*(and highlight whichever is relevant, i.e., falls, their functional decline, or depressive symptoms)* Do you feel different about asking your GP about other issues? Does your GP ever ask you about...?
**Establish the Health Care Resources that the participant has sought/engaged with to assist with their symptoms**	**The following section is split depending on whether the participant answers Yes or No to accessing services** Are your symptoms a problem for you? Have you been to see anyone to assist with your symptoms? **If Yes:** What prompted you to go?/Were you told of anyone to go to that could assist you?/What were you told? How did you feel about going? Did you feel “comfortable” or did you feel “embarrassed”? Do you have a Case Manager? (Did your Case Manager tell you about anyone you could go to?) **If No:** Were you told of anyone to go to that could assist you? Do you not know where to go?/Why do you think you did not know where to go? Do you have a Case Manager? Did your Case Manager tell you about anyone you could go to? What were you told? Did you think there was no one to help you with this? **Other:** What did they receive? What did they feel about what they received? Would they encourage others to seek assistance with their symptoms? What would they recommend? What was helpful?
**Establish what advice/changes the participant would recommend**	Now that you’ve experienced what you have over the past 6 months what is some advice that you would give someone currently in hospital?

Participants who consent will be videoed and audiotaped during their interview. Participants will be provided with a visual display of their reported depressive and anxiety symptoms for each month during their involvement in the study to assist the interviewer with ascertaining what events may have been occurring at each time period and assisting the participant to remember how they had reported to be feeling at those time periods. Ethical approval has been received to undertake audio and visual recording following consent from the participant to be involved. Audiotapes will be transcribed at the end of the study and thematic analysis will be undertaken. Strategies will be put in place to ensure credibility (in preference to internal validity), transferability (in preference to external validity), dependability (in preference to reliability), and confirmability (in preference to objectivity). [83] Visual recordings will be edited to create a short recording that may be used for future patients prior to their discharge home from hospital to hear first person what factors may impact on their transition home and what factors may assist with this transition to enable positive functional, physical and mental outcomes.
